# Current Opinion on Nanotoxicology

**DOI:** 10.1186/2008-2231-20-95

**Published:** 2012-12-15

**Authors:** Ali Pourmand, Mohammad Abdollahi

**Affiliations:** 1 Department of Emergency Medicine, George Washington University Medical Center, 2150 Pennsylvania, Ave NW, Suite 2B, Washington, DC, 20037, USA; 2 Department of Toxicology and Pharmacology, Faculty of Pharmacy and Pharmaceutical Sciences Research Center, Tehran University of Medical Sciences, Tehran, 1417614411, Iran

**Keywords:** Current opinion, Nanotoxicology, Nanotechnology

## Abstract

Nanotechnology is one of the premiere technologies available today, having expanded both as field of scientific study and in the public consciousness. Despite this growth, the drawbacks, limitations and potential safety hazards associated with the incorporation of nanotechnology into existing industries are still being learned. The noticeable point is that there is no enough data available yet to analyze global use of nanotechnology from a meta-perspective. Three challenges can be defined in light of nanotoxicology. One, materials that might prove to be significantly toxic must be identified. Two, a system for the categorization of NP materials must be codified and made available to toxicologists. Third, a better understanding of nanoparticles biological interactions must be obtained, in order to make the best use of the first two goals. For all three, it must be remembered that research standards need to be developed for the gathering of data on the nanoscale, as that level is where the NPs and the patient’s biosystems will be interacting.

As requiring toxicologists to become nanotechnology experts would not be feasible, to properly incorporate the care of nanotoxicity into the existing medical framework, a range of experts across multiple fields of study must work in close synchronization. The focus needs to be on mechanism-driven research to ensure a solid scientific foundation for the assessment of NP and their role in healthcare.

## 

Nanotechnology is one of the premiere technologies available today, having expanded both as field of scientific study and in the public consciousness. Despite this growth, the drawbacks, limitations and potential safety hazards associated with the incorporation of nanotechnology into existing industries are still being learned. The noticeable point is that there is no enough data available yet to analyze global use of nanotechnology from a meta-perspective.

The conventional definition of a nanoparticle (NP) is a material or materials with two or three dimensions, between 1 and 100 nm and possessing strange properties related to the size, shape or chemical composition of the material. The term nanotechnology can be defined as “the manipulation, measurement, precision, modeling, or manufacture of sub-100 nanometer scale matter” [1]. Today, over 800 consumer products are estimated to incorporate nanoparticles [2,3].

Of particular interest to the medical field is the potential advantage of nanotechnology in both diagnostic and therapeutic approaches. The ability to operate at the cellular or intercellular level could lead to dramatic leaps in the patient care. As of this writing, NPs are being developed to meet a variety of needs, however very few NPs have made it out of the testing phase. Over the last five years, production of NPs has increased five-fold. The current approximation is that more than 580 companies over 30 countries manage and produce some 1,317 NP-based projects, from textiles to wound dressings [2]. Some scientists have speculated that nanotechnology is growing because of recognition that a targeted, cell-by-cell approach brings greater benefits than an undifferentiated approach [4].

However, there are significant concerns associated with the use of NPs. The primary concerns are with how easily NPs can penetrate cell membranes. When compared to non-NPs, NPs can more easily pass into various biological structures, creating a potentially toxic effect. On a macro-scale, the increasing presence of NPs on an environmental level may also have negative consequences, however, the current knowledge is not enough to reach a convincing consensus. For instance, NPs designed to act as chemical catalysts for particular chemical reactions, might, due to their physical structure have undesirable effects in the human body. In one particular case, the respiratory system was harmed by NPs [5-7]. With these concerns, a growing body of professionals is seeking development of toxicology screenings that specifically target NPs [8,9].

Categorizations of NPs tend to fall into carbon-based NPs, i.e. fullerenes and carbon nanotubes, and inorganic NPs, ones based on the metal oxides (iron oxide, cerium oxide, titanium dioxide, silicon dioxide), metals (gold and silver), and semiconductor NPs or so-called quantum dots [10]. All of the above could have hazardous effects but a comprehensive analysis of each NP class has yet to occur. NPs production can be divided into four major steps such as gas-phase, vapor deposition, colloidal and attrition, and each step could cause toxicity by gastrointestinal, pulmonary or skin exposure [11].

The danger that NPs pose is related to their very nature. Due to their miniscule size, NPs can collect nearly anywhere in a biological system. Whether a person absorbs NPs in the process of taking nano-based drugs, through topical skin applications or by inadvertent inhalation of NPs makes no difference when considering the potential harm due to nanotoxicity. The toxic effects might be compounded in cases involving NPs deliberately manufactured for use in industry as those NPs might promote harmful chemical reactions once in the body.

Specifically, the small scale of NPs allow them to bypass the usual barriers to cell uptake and can enter sensitive tissues such as bone marrow, lymph nodes, spleen and the heart by way of blood circulation or lymphatic channels [12]. Of course, such entry depends on the chemical makeup of the NPs. When endocytosis occurs with a foreign matter such as NPs, there is a risk of inflammatory activity, sometimes pro-oxidant, sometimes antioxidant [12,13]. There is evidence that the body produces an oxidative stress response, which, when coupled with mitochondrial distribution may result in some disadvantages that remain to be clarified. NPs at the scale of biological macromolecules could easily hybridize, as several features of certain NPs give them advantaged when compared to biological molecules. For instance, NPs introduced to the body through sunscreen pass through the skin due to their small size, avoid neutralization by the immune system due to their composition, and their tendency to produce free radicals have concerned experts who fear that if sunscreen NPs prove toxic, they might be impossible to flush out of the body [14]. Other dangers include protein degradation brought on by the large surface area of NPs compared to the small surface area of larger particles [15], which can be observed in various processes with taking DNA macromolecules and using them to solubilize highly hydrophobic single-walled carbon nanotubes [16]. These carbon nanotubes can induce DNA strand breaks due to a greater amount of intrusion into the cell nuclei, or even create micronuclei [17].

Dosing also becomes a greatly complicated affair on the nanoscale, with the potential benefits being weighted against the danger of exposing a compromised organism to NPs [12]. There are many aspects of medicinal NPs use that must be defined, such as the effects of the NP’s size, shape and chemical composition. Furthermore, questions exist as to how long a NP dose remains in the body. Are they eventually flushed out of a particular cell? If so, how long does that process take, from start to finish? When the body absorbs NPs for therapeutic purposes, do they simple provide an enhanced version of the effects seen in large particles or are the NPs creating entirely new effects, with the added danger of unforeseen side effects [18,19]? And what of “antigenic challenges”? Could engineered NPs diminish the function of the immunological apparatus? Can the effects of NPs be predicted with any type of accuracy? Or precise measurements taken at that scale? And in the case of adverse effects, the small scale of NPs means NPs could affect organs very distant from their point of entry. Even the use of targeting ligands applied to the surface of NPs to ensure delivery of a nanodevice to a specific anatomical location or cell type. One case to focus on is the use of flexible hydrophilic polymers, specifically those incorporating polyethylene glycol (PEG), which limit opsonization of the NPs and give them opportunity to remain in circulation for longer time periods [20]. Due to the fact that the NPs remained circulating, they reached various additional structures and, depending on the type of NP, build up in those new structures. An example comes from the early days of research of the biodistribution of model polystyrene NPs, which were coated with poloxamers. These elements attach to particle surfaces via their hydrophobic areas, and the hydrophilic sections come through into the aqueous medium and these particles found to concentrate to significant extent in the bone marrow, specifically the sinusoidal bone marrow endothelium [21]. Other areas of accumulation were the brain, gut, spleen, and lymph nodes [22,23]. Obviously the potential dangers of having foreign NPs building up in the body cannot be ignored. Thus the discipline of nanotoxicology, which seeks to define and demarcate the effects caused by artificial NPs and establish the relationship between NPs and toxicity.

Up until the present day, those studying the phenomenon of nanotoxicology focused on NPs made of conventional material, TiO2, ZnO and Ag, for example, with the occasional special material such as carbon nanotubes. With the recent advances in nanotechnology however, that approach will no longer allow toxicologists to stay on top of the development and potential hazards posed by the increasingly sophisticated NPs now in production, build using multicomponent materials, hybrid materials that sit on the line between organic and inorganic, and materials designed to change their behavior based on either the environment or a received signal [24]. As such complicated NPs became more and more widely accepted, there has been a greater recognition of the lack of toxicological knowledge about these new substances [25].

Therefore, three challenges can be defined in light of this new information. One, materials that might prove to be significantly toxic must be identified. Two, a system for the categorization of NP materials must be codified and made available to toxicologists. Third, a better understanding of NPs’ biological interactions must be obtained, in order to make the best use of the first two goals. For all three, it must be remembered that research standards need to be developed for the gathering of data on the nanoscale, as that level is where the NPs and the patient’s biosystems will be interacting.

As requiring toxicologists to become nanotechnology experts would not be feasible, to properly incorporate the care of nanotoxicity into the existing medical framework, a range of experts across multiple fields of study must work in close synchronization. The focus needs to be on mechanism-driven research to ensure a solid scientific foundation for the assessment of NP and their role in healthcare.

## Competing interests

The authors have no commercial associations or sources of support that might pose a competing interest.

## Authors’ contributions

Both authors have made substantive equal contributions to the paper and read and approved the final manuscript.
